# Radiosurgery for classical trigeminal neuralgia: impact of the shot size on clinical outcome

**DOI:** 10.1186/s10194-023-01583-4

**Published:** 2023-05-11

**Authors:** Cécile Ortholan, Philippe Colin, Benjamin Serrano, Thibault Bouet, Nicolas Garnier, Maud le Guyader, Regis Amblard, Rémy Villeneuve, Stéphane Chanalet, Haiel Alchaar, Eric Bozzolo, Michel Lanteri-Minet, Denys Fontaine

**Affiliations:** 1 Department of Radiotherapy, Centre Hospitalier Princesse Grace, Monaco, Monaco; 2 Department of Radiation Oncology, Centre Hospitalier Princesse Grace, Monaco, 98000 Monaco; 3 Department of Medical Physics, Centre Hospitalier Princesse Grace, Monaco, Monaco; 4 Pain Clinic, Centre Hospitalier Universitaire de Nice, Université Côte d’Azur, Nice, France; 5 Department of Radiology, Centre Hospitalier Princesse Grace, Monaco, Monaco; 6 Department of Radiology, Centre Hospitalier Universitaire de Nice, Université Côte d’Azur, Nice, France; 7 FHU Inovpain, Centre Hospitalier Universitaire de Nice, Université Côte d’Azur, Nice, France; 8 INSERM/UdA, U1107, Neuro-Dol, Trigeminal Pain and Migraine, University Clermont-Auvergne, Clermont-Ferrand, France; 9 Department of Neurosurgery, Centre Hospitalier Universitaire de Nice, Université Côte d’Azur, Nice, France

**Keywords:** Trigeminal neuralgia, Radiosurgery, Shot size, Linear accelerator, Pain

## Abstract

**Background:**

This study compares the outcome of patients suffering from medically refractory classical trigeminal neuralgia (TN) after treatment with radiosurgery using two different shot sizes (5- and 6-mm).

**Methods:**

All patients included in this open, prospective, non-controlled study were treated in a single institution for TN (95 cases in 93 patients) with LINear ACcelerators (LINAC) single-dose radiosurgery using a 5-mm shot (43 cases) or 6-mm shot (52 cases). The target was positioned on the intracisternal part of the trigeminal nerve.

**Results:**

The mean Dmax (D0.035) to the brainstem was higher in the 6-mm group: 12.6 vs 21.3 Gy (*p* < 0.001). Pain relief was significantly better in the 6-mm group: at 12 and 24 months in the 6-mm group the rate of pain-free patients was 90.2 and 87.8%, respectively vs. 73.6 and 73.6% in the 5-mm group (*p* = 0.045). At 12 and 24 months post-radiosurgical hypoesthesia was more frequent in the 6-mm group: 47.0 and 58% vs.11.3 and 30.8% in the 5-mm group (*p* = 0.002). To investigate the effect of cone diameter and the dose to the brainstem on outcomes, patients were stratified into three groups: group 1 = 5-mm shot, (all Dmax < 25 Gy, 43 cases), group 2 = 6-mm shot, Dmax < 25 Gy (32 cases), group 3 = 6-mm shot Dmax > 25 Gy (20 cases). At 12 months the rates of hypoesthesia were 11.3, 33.5 and 76.0%, respectively in groups 1, 2 and 3 (*p* < 0.001) and the rates of recurrence of pain were 26.4, 16.5 and 5%, respectively, (*p* = 0.11).

**Conclusion:**

LINAC treatment with a 6-mm shot provided excellent control of pain, but increased the rate of trigeminal nerve dysfunction, especially when the maximum dose to the brainstem was higher than 25 Gy.

## Background

Trigeminal neuralgia (TN) is characterized by recurrent unilateral brief electric shock-like pains abrupt in onset and termination, which is limited to the distribution of one or more divisions of the trigeminal nerve and triggered by innocuous stimuli [1]. TN is considered to be classical when in the absence of an apparent cause other than neurovascular compression with morphological changes (typically displacement or atrophy) in the trigeminal root [1]. It is relatively rare, with an incidence of 1 to 2 /10,000 people, and affects more women than men (3:1 ratio). A pharmacological approach is the first-line treatment therapeutic option. For patients with medically refractory TN, invasive approaches can be offered: microvascular decompression, percutaneous rhizotomy or radiosurgery [2–5].

Radiosurgery has been the treatment of choice for medically refractory TN for over 50 years [6, 7]. The objective is to perform a neurolysis [8, 9] by delivering a very high dose of radiation in a single fraction, using a high precision radiation technique. Historically, patients were treated using GammaKnife (GK), with collimation of 4 mm, resulting in a single shot of 4 mm on the nerve. The results concerning the efficacy and toxicity of GK-radiosurgery are well documented [10–13]. Over the past years, radiosurgery has also been developed both with LINear ACcelerators (LINAC) [14–19] and the CyberKnife [20].

It has been demonstrated that some technical parameters are associated with the success and level of toxicity of radiosurgery, in particular the location of the target on the trigeminal nerve and the dose received by the brainstem [21–25]. However, the size of the shot is a parameter that has been little studied in the literature.

For patients treated on LINAC, functional radiosurgery is performed by using tertiary conical collimators named « cones» attached to the LINAC. The diameter of the cones can range from 4 to 7.5 mm and the cone diameter reflects the shot size. Most studies reported the outcome of patients treated with 4- or 5-mm cones [16, 26–31], whereas in the largest LINAC series the patients were treated with a 6-mm cone [15].

The influence of the shot size on outcome is not well established. One study suggested better pain relief using 5-mm cones compared to a 4-mm cone [30]. To date, there is no comparison between the 5- vs 6-mm cone. We studied and compared the outcome of two consecutive series of TN patients treated in the same institution with LINAC radiosurgery using two shot sizes: 5-or 6-mm.

## Methods

### Patients and study design

Patients treated with radiosurgery for trigeminal neuralgia in a single institution were included in a prospective database, collecting technical data, efficacy and toxicity of the radiosurgery. From this database, we extracted the data from consecutive patients treated from April 2018 to November 2021 for medically refractory classical TN and we performed an open, prospective, non-randomized study to evaluate the effect of the shot size on their outcome. From April 2018 to October 2019 all treatments were collimated by a 6-mm cone (52 procedures). From October 2019, after the commissioning of the 5-mm cone, all treatments were collimated by a 5-mm cone (43 procedures).

Classical TN was diagnosed according the International Classification of Headache Disorders [1]. Patients suffering from secondary TN (tumor in the cerebellopontine angle, arteriovenous malformation or multiple sclerosis), and classical TN with a previous history of a radiosurgery procedure were excluded.

Patients had a brain MRI to discard differential diagnoses. All patients presented with TN resistant to the maximum tolerated medical therapy, with an intensity of pain according to the Barrow Neurological Institute (BNI) score IV or V. Indication of radiosurgery was performed by a multidisciplinary team involving a neurosurgeon, a neurologist, and a radiation oncologist.

Ninety-five procedures were performed on 93 patients because two patients were treated on both sides successively during the study. The intervals between the two radiosurgical procedures were 5 and 10 months. Each side was considered as an independent case for the statistical analysis.

### Treatment preparation and planning

A brain MRI was performed before treatment, with a 3D T2 gradient echo sequence (1 mm-slice thickness images), 3D T1-weighted sequence and a vascular sequence (TOF) to visualize the trigeminal nerve, the vascular structures and to measure the prepontine cistern.

All patients were treated with a frameless technique. For landmark identification CT-Scan, patients were immobilized with a bivalve 5-point fixation BrainLab mask (Munich, Germany). The data of the scan were imported into the treatment planning system iPlanNET, BrainLab (version 4.5.3), and co-registered with MRI data. The trigeminal nerve was identified on the 3D T2 sequence. The delineated areas at risk were: brain stem, temporal lobes, vascular structures, optical system, and cochlea. All targets and areas were delineated by 2 radiation oncologists and a neurosurgeon.

**Fig. 1:**
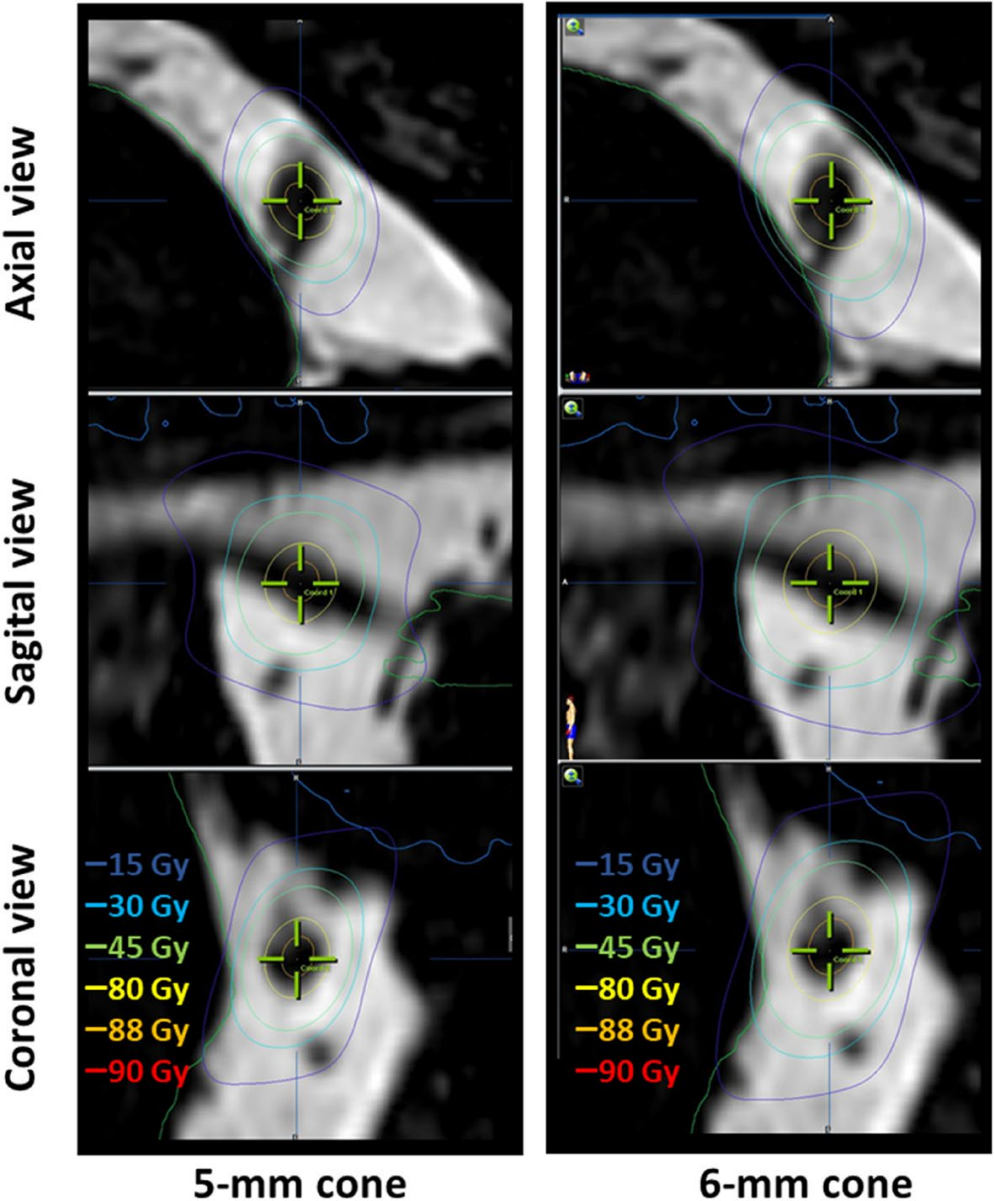
Comparison of two dosimetric plans for the same patient, using the same isocenter (8 arcs): 5-mm cone (left size) and 6-mm cone (right size). The total length of the nerve was 10.2 mm. The length of the nerve included in the isodose 45 Gy was 5.9 mm for the 5-mm cone and 6.9 mm for the 6-mm cone. The brainstem D0.035 was 13.3 Gy for the 5-mm cone and 20.2 Gy for the 6-mm cone

Dosimetry was performed on iPlanNET with an arc therapy technique. The chosen ballistic consisted in 7 to 15 non-coplanar arcs of about 100° per arc [16], with an additional cone collimation of 5 or 6 mm. The radiosurgical target was defined in the middle of the intracisternal portion of the trigeminal nerve. The dose delivered to all patients was 90 Gray (Gy) per fraction. For patients with a shorter nerve, the position of the isocenter was adjusted according to the 30% isodose line tangential to the pontine surface. Doses to the brainstem were reported as V15 and V27 (volume in cc of the brainstem receiving respectively more than 15 and 27 Gy) and maximum-point dose (Dmax) as D0.035 cc (dose delivered to 0.035 cc of the brainstem). An example of a dosimetry comparing the 5-and 6-mm cone is illustrated in Fig. 1.

### Treatment

The treatment was delivered using the Novalis Tx™ (Brainlab, Munich, Germany, Varian, Palo Alto, California, US) associating a 6-megavolt flattening filter-free Varian LINAC with mounted BrainLab accessories: tertiary conical collimators, 6-degree of freedom couch, ExacTrac BrainLab imaging system. All patients were treated in the outpatient unit.

The precision of the positioning was obtained using the ExacTrac BrainLab system using two stereoscopic images including the base of the skull merged with the digital reference images obtained with the dosimetric scan. During the session, each table rotation corresponded to a given arc checked with the ExacTrac system. The deviation tolerance was < 0.5 mm and < 0.5° in all axes. Above this threshold, the position of the patient was systematically corrected by the robotic couch. Radiation delivery was 1400 MU/minute.

### Quality assurance

Extensive quality assurance methods were followed. Commissioning of the LINAC and collimation cones were performed using ion-chamber, diodes, MicroDiamond and EBT3 radiochromic films. The testing of the geometric and dosimetric accuracy including imaging of the entire treatment process as End-to-End was examined using an anthropomorphic head phantom. The resulting root-mean square geometric error was less than 1 mm; the error for the absorbed dose was less than 4% for the 5-mm cone and less than 3% for higher sizes. Winston-Lutz tests for daily quality assurance were performed. The results showed a radius error less than 0.45 mm for 5- and 6-mm cones.

### Evaluation of efficacy and patients’ follow-up

Following radiosurgery, all patients underwent follow-up evaluation at 1, 6 and 12 months, then once a year or earlier if the pain came back of a side effect occurred. Pain related to TN was assessed according to the Barrow Neurological Institute (BNI) pain intensity scale 2.0 [32]: Class I, no trigeminal pain, no medication; Class II, occasional pain, not requiring medication; Class IIIa, no pain with continued medication; Class IIIb, pain controlled with medication; Class IV, some pain, not adequately controlled with medication; and Class V, severe pain, no pain relief. A successful outcome was defined for classes I to IIIa. Classes IIIb-IV-V were considered as treatment failure. Pain recurrence was defined as a change from class I-IIIa to higher BNI classes.

The following side effects were investigated at each visit: hypoesthesia, paresthesia, neuropathic pain, alteration of corneal reflex and jaw dysfunction. The BNI facial hypoesthesia scale was used to assess patients’ radiation-induced facial hypoesthesia: Grade I no facial hypoesthesia; grade II mild facial hypoesthesia, not bothersome; grade III somewhat bothersome facial hypoesthesia; grade IV very bothersome facial hypoesthesia.

### Statistical analysis

Statistical analysis was done in May 2022. Chi-square test was used to compare the categorical time and t-test to compare the means of groups of patients. The number of pain-free patients was assessed using a Kaplan-Meier curve, and defined by the interval between the time of treatment by radiosurgery and the time of pain recurrence. Patients presenting with initial treatment failure were pooled with recurrent patients. In this case, the time of recurrence was defined at 1 month. Rate of hypoesthesia- or paresthesia-free patients were assessed using Kaplan-Meier rates, and defined by the interval between the time of treatment with radiosurgery and the time of occurrence of the side effect. The hazard ratio and log-rank test were used to compare groups of patients. Analyses were performed with the SPSS 16.0 software (SPSS Inc., Chicago, Ill). All tests were bilateral and a *p* value < 0.05 was considered statistically significant.

## Results

### Population characteristics

Population characteristics according to the cone diameter are illustrated in Table 1. Fifteen patients had previously received an invasive procedure: single percutaneous thermal rhizotomy (TRZ) for 11 cases, two TRZ for 1 case, microvascular decompression (MVD) only for 1 case, TRZ and MVD for 1 case. Doses to the brainstem are summarized in Table 2. The maximum dose to the brainstem, V15 and V27 were significantly higher in the 6-mm group than in the 5-mm group.

**Table 1:** Characteristics of the population according to the treatment group

	5-mm cone	6-mm cone	All	*P* value
Number of cases	43	52	95	
Gender				
Men	20 (42.6%)	27 (57.4%)	47	*p* = 0.60
Women	23 (49.7%)	25 (52.1%)	48	
Age				
Mean	72.6	70.6	71.5	*p* = 0.31
Min–max	[47–95]	[31–92]	[31–95]	
Side				
Right	22 (41.5%)	31 (58.5%)	53	*p* = 0.41
Left	21 (50%)	21 (50%)	42	
Affected branches				
One branch	16 (38.1%)	26 (61.9%)	42	*p* = 0.41
Two branches	17 (48.6%)	18 (51.4%)	35	
Three branches	10 (55.6%)	8 (44.4%)	18	
Neurovascular conflict				
Yes	15 (45.5%)	18 (54.5%)	33	*p* = 0.98
No	28 (45.2%)	34 (54.8%)	62	
Duration of pain (years)				
Mean	7.3	9.7	8.5	*p* = 0.06
Min–max	[1–30]	[0–44]	[0–44]	
Previous invasive procedure				
Yes	7 (46.7%)	8 (53.3%)	15	*p* = 0.90
No	36 (45%)	44 (55%)	80

**Table 2:** Comparison of the dose to the brainstem according to treatment group. V15 and V17 = volume of brainstem receiving more than 15 Gy and 17 Gy, in cc. Dmax = dose received by 0.035 cc of the brainstem (D0.035)

	5-mm cone	6-mm cone	All	*P* value
Dmax (mean)	12.6 Gy	21.3 Gy	17.3 Gy	*p* < 0.001
Dmax (median)	13.3 Gy	20.4 Gy	15.0 Gy	
[Min–max]	[5.3–16.6]	[10.8–31.5]	[5.3–31.5]	
V15 (mean)	0.021 Gy	0.108 Gy	0.068 Gy	*p* < 0.001
[Min–max]	[0.000–0.057]	[0.004–0.231]	[0.000–0.231]	
V27 (mean)	0.002 Gy	0.020 Gy	0.012 Gy	*p* < 0.001
[Min–max]	[0.000–0.010]	[0.000–0.057]	[0–0.057]	

### Median follow-up

The median follow-up was 23.5 months [6.0–46.8 months]: 15.8 months in the 5 mm group [6.0–29.4] and 32.0 in the 6 mm group [6.0–46.8]. At final follow-up, one patient had died (unrelated to the radiosurgery).

### Efficacy of initial pain relief and duration of the clinical response

After radiosurgery, 87 out of 95 (91.5%) cases were BNI class I to IIIa (Table 3). The success rates were 96.1% in the 6-mm group and 86.0% in the 5-mm group (*p* = 0.08). The percentage of patients in BNI class I was significantly higher in the 6-mm group: 73.0 vs 53.5% (*p* = 0.047). For the patients with initial treatment success, the mean delay between radiosurgery and best pain relief was 3.3 months [0.0–16.0]: 2.4 months in the 5-mm group and 3.5 months in the 6-mm group (*p* = 0.08).

**Table 3:** Comparison of the Barrow Neurological Institute (BNI) pain intensity score according to the treatment group. BNI score: Class I, no trigeminal pain, no medication; Class II, occasional pain, not requiring medication; Class IIIa, no pain with continued medication; Class IIIb, pain controlled with medication; Class IV, some pain, not adequately controlled with medication; and Class V, severe pain, no pain relief

	5-mm cone	6-mm cone	All
Number of cases	43 (%)	52 (%)	95 (%)
BNI score			
I	23 (53.5%)	38 (73.0%)	61 (64.2%)
II	7 (16.3%)	6 (11.6%)	13 (13.7%)
IIIa	7 (16.3%)	6 (11.6%)	13 (13.7%)
IIIb	1 (2.3%)	2 (3.8%)	3 (3.1%)
IV	0 (0%)	0 (0%)	0 (0%)
V	5 (11.6%)	0 (0%)	5 (5.3%)
Treatment success (Class I-IIIa)	37 (86.1%)	50 (96.1%)	87 (91.2%)

Among the 87 patients with initial pain relief, 10 (11.5%) reported pain recurrence, after a median time of 6.9 months [3.9–34.1]: 5/37 (13.5%) in the 5-mm group (median time 6.6 months) and 5/50 (10.0%) in the 6-mm group (median time 7.5 months).

**Fig. 2:**
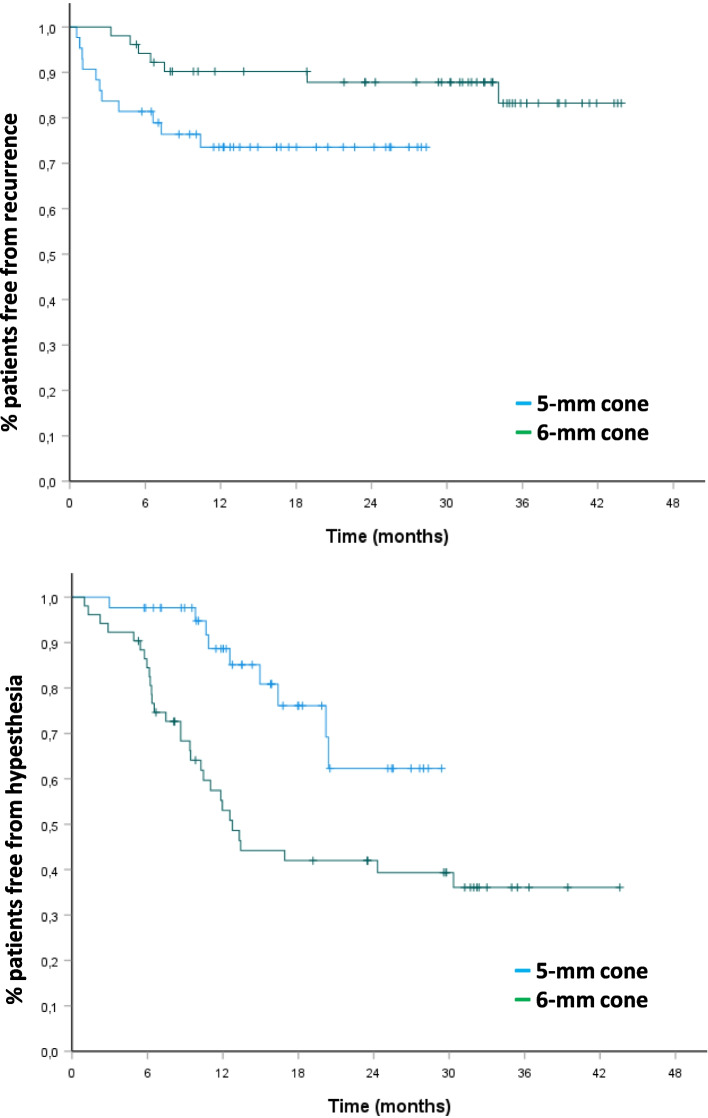
Probability of treatment success and facial hypoesthesia. The number of pain-free patients and hypoesthesia-free patients was evaluated with the Kaplan-Meier method, and defined as the interval between the date of treatment with radiosurgery and the date of recurrence of pain or hypoesthesia. Patients presenting with initial treatment failure were pooled with recurrent patients (in this case, the date of recurrence was defined at 1 month). The overall percentage of patients free from pain was 82.8% at 12 months and 81.1% at 24 months. The percentage of patients free from pain (class I to IIIa) at 12 and 24 months was 73.6 and 73.6%, respectively in the 5-mm group vs 90.2 and 87.8% in the 6-mm group (p = 0.045). The percentage of hypoesthesia was 11.3 and 30.8% at 12 and 24 months, respectively in the 5-mm group and 47.0 and 58% in the 6-mm group (*p* = 0.002)

Among the patients alive at the end of follow up, 18 had treatment failure (initial failure or delayed recurrence): 11/43 in the 5 mm group and 7/51 in the 6-mm group. At 12 and 24 months, the successful outcome rates (class I to IIIa) were 82.8 and 81.1%, respectively. According to the shot size, the success rates at 12 and 24 months were 73.6 and 73.6% in the 5-mm group vs 90.2 and 87.8% in the 6-mm group, *p* = 0.045 (Fig. 2).

### Adverse effects

Side effects are detailed in Table 4. Forty patients presented with new, persistent facial hypoesthesia. Hypoesthesia occurred in one, 2 or 3 branches in 11, 14 and 15 patients, respectively. Hypoesthesia (grades II-IV) was present in 31.3% of the patients 12 months after radiosurgery and in 49.4% at 24 months. Hypoesthesia was more frequent in the 6-mm group: 11.3% and 30.8% at 12 and 24 months respectively in the 5-mm group vs. 47.0% and 58% in the 6-mm group (*p* = 0.002). When considering only bothersome hypoesthesia, namely grades III and IV, hypoesthesia did not significantly differ across groups: Kaplan-Meier rates at 12 and 24 months were 8.4 and 27.5% in the 5-mm group vs. 20.2 and 24.7% in the 6-mm group, (*p* = 0.52).

**Table 4:** Comparison of the side effect according to the treatment group. Barrow Neurological Institute (BNI) facial hypoesthesia scale: Grade I no facial hypoesthesia, grade II mild facial hypoesthesia, not bothersome, grade III somewhat bothersome facial hypoesthesia, grade IV very bothersome facial hypoesthesia

	5-mm cone	6-mm cone	All
Number of cases	43	52	95
Cases with at least one grade ≥ 2 toxicity	15 (34.9%)	37 (71.1%)	52 (54.7%)
Hypoesthesia			
I (no hypoesthesia)	33 (76.7%)	22 (42.3%)	55 (57.9%)
II	3 (7.0%)	17 (32.7%)	20 (21.1%)
III	7 (16.3%)	11 (21.2%)	18 (18.9%)
IV	0 (0%)	2 (3.8%)	2 (2.1%)
Paresthesia			
Yes	10 (23.2%)	17 (32.7%)	27 (28.4%)
No	33 (76.8%)	35 (67.3%)	68 (71.6%)
Neuropathic pain			
Yes	0 (0%)	2 (3.8%)	2 (2.1%)
No	43 (100%)	50 (96.2%)	93 (97.9%)
Jaw dysfunction			
Yes	2 (4.7%)	11 (21.1%)	13 (13.7%)
No	41 (95.3%)	41 (78.9%)	82 (86.3%)

Persistent facial paresthesia occurred in 29 patients. Kaplan-Meier rates of paresthesia at 12 and 24 months were 21.8 and 30.8% in the 5-mm group vs. 26.7 and 35.9% in the 6-mm group, *p* = 0.70. One patient in the 5-mm group and 5 in the 6-mm group had bothersome paresthesia.

The percentage of patients with jaw dysfunction was significantly higher in the 6-mm group (*p* = 0.02) (Table 4). In the 6-mm group, 2 patients developed neuropathic pain and 2 patients had an alteration in the corneal reflex.

### Influence of dose to the brainstem on outcome

**Fig. 3:**
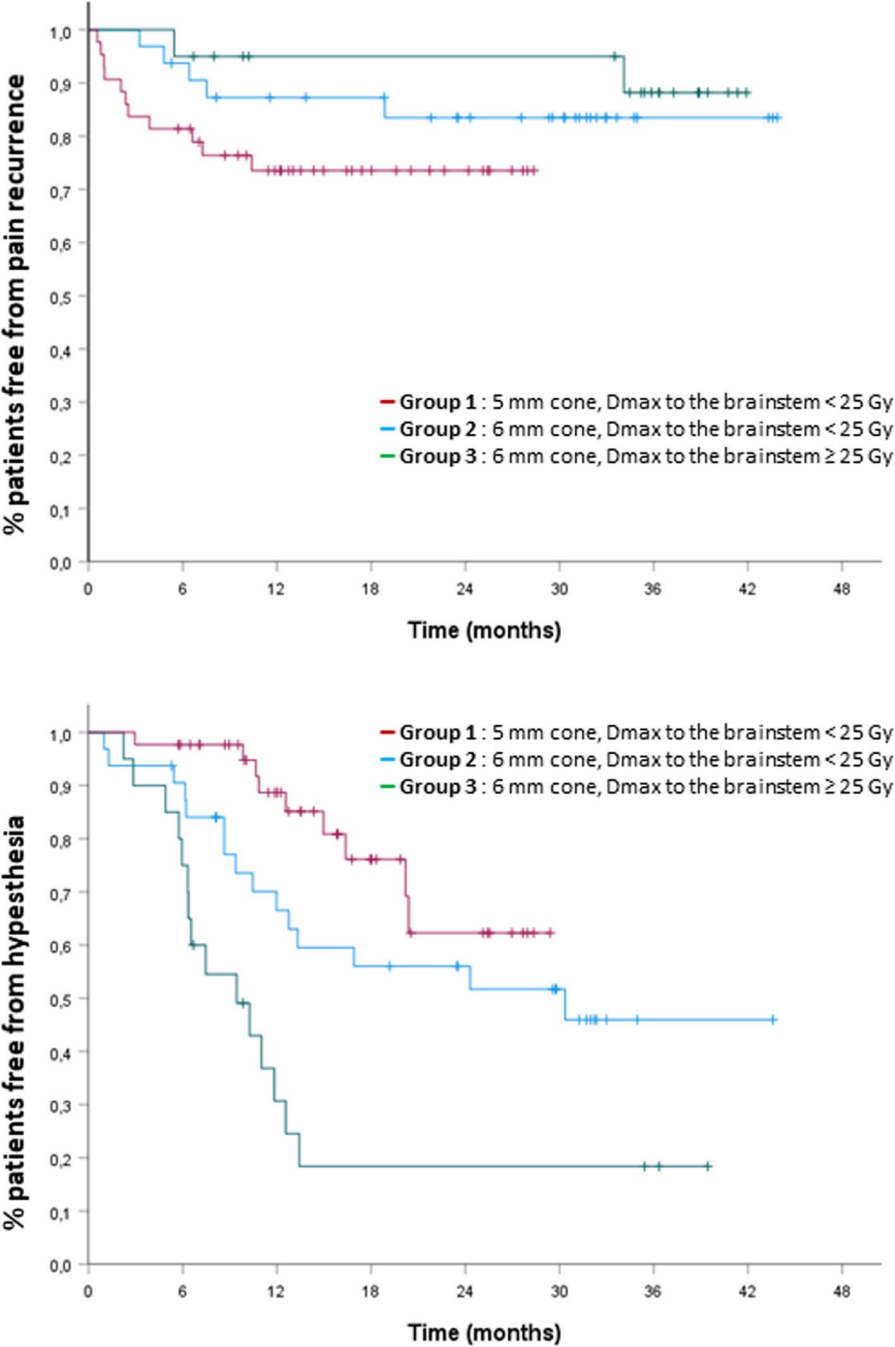
The probability of treatment success and facial hypoesthesia according to the size of the shot and maximum dose (D0.035) to the brainstem. Groups 1 = patients treated with the 5-mm shot, (all maximum dose to the brainstem < 25 Gy, 43 patients), groups 2 = patients treated with the 6-mm shot, maximum dose to the brainstem < 25 Gy (32 patients), group 3 = patients treated with the 6-mm shot with a maximum dose to the brainstem ≥ 25 Gy (20 patients). The level of 12 months hypoesthesia was 11.3, 33.5 and 76.0%, respectively, *p* < 0.001 in each group. The level of recurrence of pain at 12 months was 26.4, 16.5 and 5%, respectively, *p* = 0.11

To investigate the effect of cone diameter and the dose to the brainstem on outcomes, patients were stratified into three groups: group 1 = patients treated with the 5-mm cone with a maximum dose to the brainstem < 25 Gy (43 patients), group 2 = patients treated with the 6-mm cone with a maximum dose to the brainstem < 25 Gy (32 patients), group 3 = patients treated with the 6-mm cone with a maximum dose to the brainstem ≥ 25 Gy (20 patients). The risk of hypoesthesia and recurrence differed across groups. At 12 months, the rates of hypoesthesia were 11.3, 33.5 and 76.0%, respectively in groups 1, 2 and 3 (*p* < 0.001) and the rates of recurrence of pain were 26.4, 16.5 and 5%, respectively, (*p* = 0.11) (Fig. 3).

## Discussion

Here, we report on a large series of TN patients treated with high dose LINAC-based radiosurgery, using two different shot sizes, namely 5 and 6-mm. We observed better pain control in the 6-mm group (96% of BNI classes I-IIIa) than in the 5-mm group (86%) but at the expense of more sensory disturbance. To our knowledge, this study is the first one comparing 5 and 6-mm shot in LINAC radiosurgery for TN. Our comparative study was not randomized but the two groups of patients were treated using 2 different cone diameters according to the period of time. However, the two groups did not differ significantly in term of demography, TN severity, duration, topography and previous treatments (Table 1), which allowed the respective outcomes of 5- and 6-mm shot LINAC radiosurgery for TN to be compared.

The overall efficacy and complications observed in our study were concordant with the literature. In a comprehensive review, Tuleasca et al. [7] reported a mean initial control of pain of 84.8% with GK and 87.3% with LINAC. The delayed adverse effect of hypoesthesia for patients treated with LINAC ranged from 11.4 to 49.7%. In the 5-mm group, our results compared favorably to the literature, although our study is limited by its short follow-up, considering the natural history of TN and risk of recurrence that increases with time. In the 6-mm group, we report an excellent level of control of pain compared to literature, whereas our rate of hypoesthesia compared unfavorably with the current data [7]. This negative correlation between hypoesthesia and treatment success has previously been described [11] and may be explained by technical considerations. Indeed, it has been demonstrated that the outcome of TN radiosurgery was influenced by interdependent technical parameters such as total dose to the target, shot size, length of treated nerve, dose delivered to the brainstem, location of the target within the nerve and volume of the proximal nerve irradiated [6, 13, 21–26, 30, 33–35].

The influence of the shot size on outcome was recently discussed by Kienzler et al. [30] in a published a series of 234 patients treated on a LINAC: 97 patients were treated with a 4-mm cone and 137 patients with a 5-mm cone using a dose of 90 Gy. The target was more proximal than ours (50% isodose surface tangential or sightly tangential to the brainstem). The initial rate of control of pain (BNI I-III) was better in the 5-mm group and the difference between groups lasted, with a rate at 24 months of 89.9 vs. 77.8%, respectively (*p* = 0.02). The authors reported no relationship between hypoesthesia and cone size.

It has been established that treating a longer portion of the trigeminal nerve increased the risk of late hypoesthesia but had no effect on control of pain. Koca et al. [36] evaluated, in 21 consecutive patients, LINAC radiosurgery (70 Gy) encompassing the whole nerve (90% isodose fully enveloped the nerve). Complete pain relief was achieved in 90.5% of the patients and 42.9% presented with new-onset hypoesthesia at the last follow-up. Another series of 101 patients treated with a GK (dose 75 Gy) showed that the combined use of 4- and 8-mm collimators did not improve control of pain when compared with a single 4-mm collimator with an equivalent maximum dose [37]. Flickinger et al. [33] randomized 87 patients to undergo retrogasserian GK radiosurgery (75 Gy maximal dose with 4-mm diameter collimators) using either one or two isocenters (resulting in a longer portion of the treated nerve). The pain relief was identical in both groups, whereas the rate of sensory disturbance significantly correlated with the nerve length irradiated.

Increasing the dose to the brainstem seems to confer both better pain control and higher rate of trigeminal dysfunction [23]. A higher dose to the brainstem may result either from targeting a more proximal portion of the nerve, from a shorter length of the nerve, or both, which results in a higher level of exposure to sensory fibers [21, 25, 35]. The best portion of the nerve to target remains controversial: most authors suggest that more proximal targets induce more hypoesthesia but better pain relief [6, 21, 22, 25], whereas others did not find any relationship between the position of the target and outcome [29]. An optimal target should balance the ratio between efficacy and toxicity. In a series of 106 patients treated with GK targeting the dorsal root entry zone (90 Gy), Hung et al. observed that facial hypoesthesia correlated with the nerve length and with the targeting ratio, namely the ratio between nerve length and targeting length. They concluded that for patients with longer nerves (> 11 mm), proximal targeting (targeting ratio < 36%) increased the rate of hypoesthesia [24]. Another large series of 329 TN patients treated on a LINAC with the 6-mm cone (90 Gy) was recently published by Debono et al., with a more proximal target on the plexus triangularis. The authors reported a low rate of bothersome (grades III-IV) hypoesthesia (5 vs 24.7% in the 6-mm group in our series) and hypothesized that this low rate might be related to a lower dose to the brainstem [15]. In our study, we observed that the combination of a 6-mm shot and a high dose to the brainstem (≥ 25 Gy) was associated with less recurrence of pain (5%) but more hypoesthesia (76% at 12 months), whereas the 5-mm shot was associated with more recurrence (26.4%) and less hypoesthesia (11.3%). Together, the data suggests that for patients treated with the 6-mm shot, a more distal target, lowering the dose to the brainstem, may lead to a better compromise between pain control and hypesthesia.

## Conclusions

LINAC radiosurgery using a 5-mm shot provided an excellent clinical outcome for refractory TN patients, which is consistent with the literature. However, the same LINAC radiosurgery using 6-mm shot achieved a better pain relief, but increased the risk of sensory disturbance, especially when the maximum dose to the brainstem was higher than 25 Gy. For patients treated with the 6-mm shot, more distal targets on the trigeminal nerve, namely closer to the plexus triangularis should be preferred, to decrease exposure of the brainstem to radiation. The maximum dose to the brainstem should be less than 25 Gy for these patients. The choice of the shot size (5- or 6-mm) should be based on a customized collegial decision including the patient, taking into consideration the risk of recurrence of pain, the possibility of other salvage treatments and the acceptability of side effects. Patients treated with the 6-mm shot and proximal target should be informed of the higher risk of trigeminal dysfunction.

## Data Availability

The data that support the findings of this study are available from Dr Cécile Ortholan but restrictions apply to the availability of these data, which were used under license for the current study, and so are not publicly available. Data are however available from the authors upon reasonable request and with permission from Dr Cécile Ortholan, Dr Michel Lanteri-Minet and Pr Denys Fontaine.

## Abbreviations

TN: Trigeminal neuralgia
GK: GammaKnife
LINAC: Linear accelerator
Gy: Gray
Dmax: Maximum-point dose
BNI: Barrow Neurological Institute
TRZ: Single percutaneous rhizotomy
MVD: Microvascular decompression
